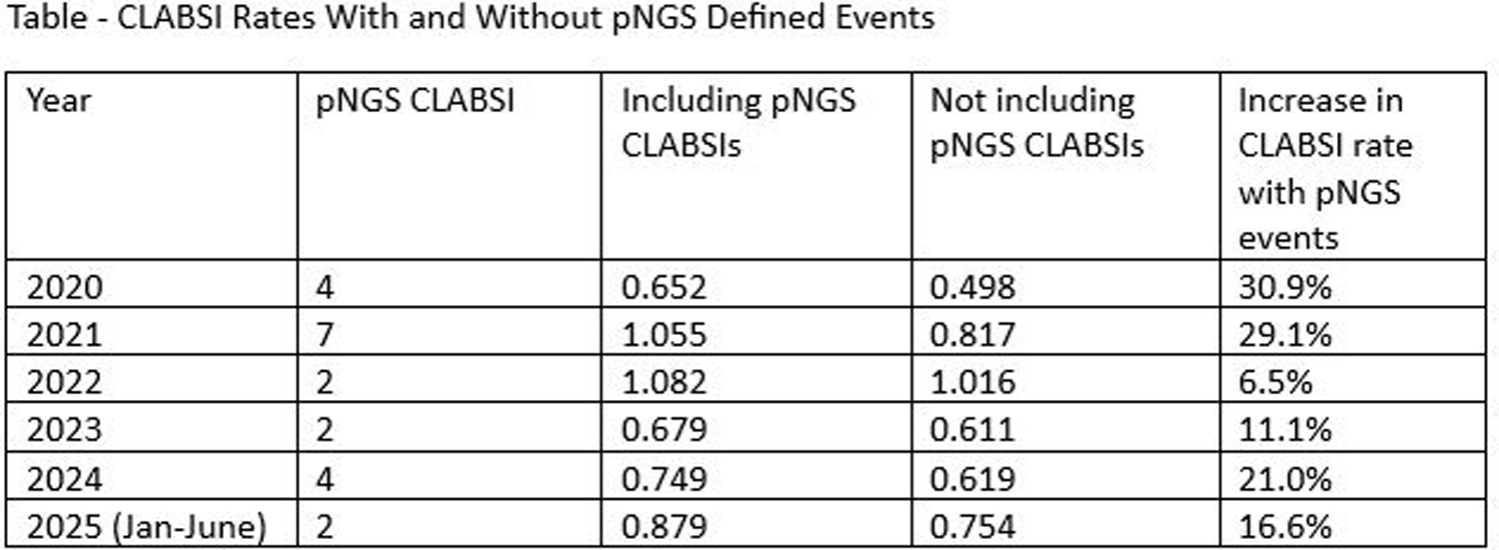# 164 Randomized Crossover Simulation Evaluation of Human-Factors–Driven PPE Ensembles for Isolation Care

**DOI:** 10.1017/ash.2026.10565

**Published:** 2026-06-23

**Authors:** Alice Pong, Megan Medina, Edmund Milder, Sondra Lintz, John Bradley, Rachel Marrs

**Affiliations:** 1 Rady Childrens Hosp San Diego; 2 Rady Children’s Hospital; 3 UC San Diego Health, Rady Children’s Health; 4 Rady Children’s Hospital San Diego; 5 Dept of Pediatrics, UCSD School of Medicine; 6 Rady Children’s Health

## Abstract

**Background:** PNGS technology to identify microbial DNA in blood, is used to diagnose infections not identified with standard cultures. The National Healthcare Safety Network (NHSN) defines central line associated bloodstream infection (CLABSI) as an NHSN defined laboratory confirmed bloodstream infection (LCBI) in the presence of an eligible central line. The LCBI definition was updated in 2020, to include pNGS as a non microbiologic test to define CLABSI, not accompanied by a standard blood culture. This study describes the adverse impact of pNGS on CLABSI rates at a pediatric hospital. **Methods:** CLABSI events attributed to pNGS results, were identified through hospital infection control records from January 2020 through June 2025. Medical charts were reviewed for blood cultures, indications for testing, prior pNGS, and changes in antimicrobial therapy based on pNGS results. CLABSIs associated with mucosal barrier injury (MBI) were not included as these are not part of nationally reported rates. **Results:** 138 eight CLABSI events were identified during this timeframe, with 21(15.2%) defined by pNGS results. CLABSI rates are shown in the Table. Of pNGS defined CLABSI events, 11 patients had sites of infection other than blood, and 6 had pNGS done only to follow up prior pNGS results. Eight children with organisms identified on pNGS were already being treated and 8 new organisms on pNGS results were considered not clinically significant and not treated. Antimicrobial therapy was given in response to pNGS results for 5 patients: a neonate with candida and ureaplasma on pNGS, a pancreatitis patient with Enterococcus avium and Staphylococcus epidermidis on pNGS, a patient started on ganciclovir due to CMV, and 2 patients treated for organisms on pNGS associated with previously identified non bloodstream infections. **Conclusion:** Inclusion of pNGS defined CLABSI events increased reported CLABSI rates by a mean 19.2% per year. Most pNGS results were associated with non bloodstream infections. The clinical significance of pNGS results is uncertain and positive results do not correlate with bacterial bloodstream infections. PNGS results should not be considered comparable to traditional blood cultures and should not be used to define CLABSI events.

**Figure:**